# Classical Machine Learning Versus Deep Learning for the Older Adults Free-Living Activity Classification

**DOI:** 10.3390/s21144669

**Published:** 2021-07-07

**Authors:** Muhammad Awais, Lorenzo Chiari, Espen A. F. Ihlen, Jorunn L. Helbostad, Luca Palmerini

**Affiliations:** 1 Department of Computer Science, Edge Hill University, Ormskirk L39 4QP, UK; 2 Department of Electrical, Electronic, and Information Engineering Guglielmo Marconi, University of Bologna, 40126 Bologna, Italy; lorenzo.chiari@unibo.it (L.C.); luca.palmerini@unibo.it (L.P.); 3 Health Sciences and Technologies Interdepartmental Center for Industrial Research, University of Bologna, 40126 Bologna, Italy; 4 Department of Neuromedicine and Movement Science, Faculty of Medicine and Health Sciences, Norwegian University of Science and Technology, N-7493 Trondheim, Norway; espen.ihlen@ntnu.no (E.A.F.I.); jorunn.helbostad@ntnu.no (J.L.H.)

**Keywords:** physical activity classification, older adults, classical machine learning, deep learning, free living, wearable sensors

## Abstract

Physical activity has a strong influence on mental and physical health and is essential in healthy ageing and wellbeing for the ever-growing elderly population. Wearable sensors can provide a reliable and economical measure of activities of daily living (ADLs) by capturing movements through, e.g., accelerometers and gyroscopes. This study explores the potential of using classical machine learning and deep learning approaches to classify the most common ADLs: walking, sitting, standing, and lying. We validate the results on the ADAPT dataset, the most detailed dataset to date of inertial sensor data, synchronised with high frame-rate video labelled data recorded in a free-living environment from older adults living independently. The findings suggest that both approaches can accurately classify ADLs, showing high potential in profiling ADL patterns of the elderly population in free-living conditions. In particular, both long short-term memory (LSTM) networks and Support Vector Machines combined with ReliefF feature selection performed equally well, achieving around 97% F-score in profiling ADLs.

## 1. Introduction

Physical inactivity is classified as one of the four leading factors causing mortality. It contributes to 6% of worldwide deaths [1]. It is considered one of the primary causes of life-threatening diseases, since inactive lifestyles can trigger the prevalence of health conditions such as breast cancer, colon cancer, heart disease, and diabetes [1]. On the other hand, physical activity (PA) is essential to improve the quality of life and functional health of the elderly population. Promoting physical activity in daily life can improve physical and mental health, particularly at an older age [2,3]. A study by the European Commission suggested that the elderly population in the EU is expected to increase above 150 million by 2060 [4], and that this will require health and public infrastructures to take extraordinary measures to accommodate the ever-increasing elderly population and to promote healthy ageing and wellbeing. Therefore, there is a clear need to develop feasible and sustainable methods that can potentially monitor the activities of daily living (ADLs) of the elderly population. By capturing accelerations and angular velocities, wearable inertial measurement units (IMU) can provide unobtrusive, reliable, and low-cost measurement of ADLs.

Several wearable IMU-based physical activity classification (PAC) systems have been developed in the past. They can be broadly categorized into two primary machine learning (ML) branches, i.e., classical ML and deep learning.

The processing pipeline of classical ML-based PAC systems [5,6] consists of several stages: pre-processing (e.g., denoising, filtering), feature engineering (time and frequency domain descriptors), feature selection, and classification algorithms (e.g., support vector machines (SVM) [7], decision trees [8], k-nearest neighbours [9], and artificial neural networks [10]). In the feature engineering stage, handcrafted features are extracted by relying on the domain knowledge and, sometimes, on the biomechanical characteristics of human motion. Such a process provides an acceptable level of performance to classify ADLs. However, this manual stage could lead to potentially important information being missed [11].

Conversely, deep learning [12] automatically performs feature extraction without human intervention. The deep learning algorithms, or deep neural networks (DNNs), learn complex features automatically by adding non-linearity in the feature space (which is often overlooked in handcrafted feature extraction). This approach enables the DNN to learn complex patterns from the underlying raw data streams. The performance of such DNNs depends to a high degree on various hyperparameters linked to the optimization procedure and on the internal architecture of the DNN. The commonly used deep learning algorithms comprise (but are not limited to) convolutional neural networks (CNNs), recurrent neural networks (RNNs), and long short-term memory (LSTM) networks [13]. The existence of CNN [14] and RNN [15] deep learning algorithms goes back to the 1990s; however, these algorithms were unable to gain much attention due to the unavailability of powerful computational resources and a sufficient amount of data. More recently, deep learning algorithms have seen unprecedented levels of participation in almost every domain, ranging from digital health [16], energy forecasting [17], autonomous cars [18], and speech recognition [19], to the finance industry [20], due to the availability of high-performance computing resources and the presence of a growing amount of labelled data to train machine learning models. These deep learning algorithms have also gathered significant attention from the research community working in the domain of PAC. Therefore, several deep learning-based PAC systems have been developed in the last few years to classify ADLs [21,22,23,24,25,26,27,28]. However, these deep learning-based systems were mostly trained and tested on young adults [22,26,29,30], while very few systems have been developed for older adults focusing on PAC [31] and falls [32].

None of the PAC systems developed so far on older adults’ data have been validated in free-living conditions. In a previous benchmark study [33], we highlighted that ADLs performed in free-living conditions are different from those performed in laboratory settings or constrained environments. The performance of existing classical ML-based PAC systems highly deteriorates when tested in free-living conditions. This is because ADLs performed in a laboratory-based environment lack ecological validity and differ from those performed in free-living conditions. Therefore, PAC systems designed for elderly populations in free-living conditions should ideally be trained and tested on data recorded in the same age group and setting. The benchmark study [33] also highlighted that the performance of such PAC systems is highly dependant on several factors: the dataset, the number and placement of sensors, the feature set, the feature extraction window size, and the classifier.

In light of this, we previously developed a classical ML-based PAC system for older adults to classify their ADLs in free-living conditions [34]. The current work continues our previous efforts by developing deep learning-based PAC systems which have never been trained and/or tested on the elderly population, to the best of our knowledge. Using a fully validated free-living dataset of older adults’ ADLs, we aim to compare classical ML-based PAC systems and deep learning-based PAC systems. Recently, only a couple of studies [35,36] have investigated the performance of classical ML versus deep learning algorithms. Nevertheless, these studies focused on young adults performing ADLs in a laboratory-constrained environment.

In summary, the objectives of the current study are:

1. To develop a physical activity classification (PAC) system for an older population in free-living conditions using a deep learning approach.
2. To compare the performance between classical machine learning-based PAC system and deep learning-based PAC system.

## 2. Materials and Methods

### 2.1. Dataset

**Figure 1:**
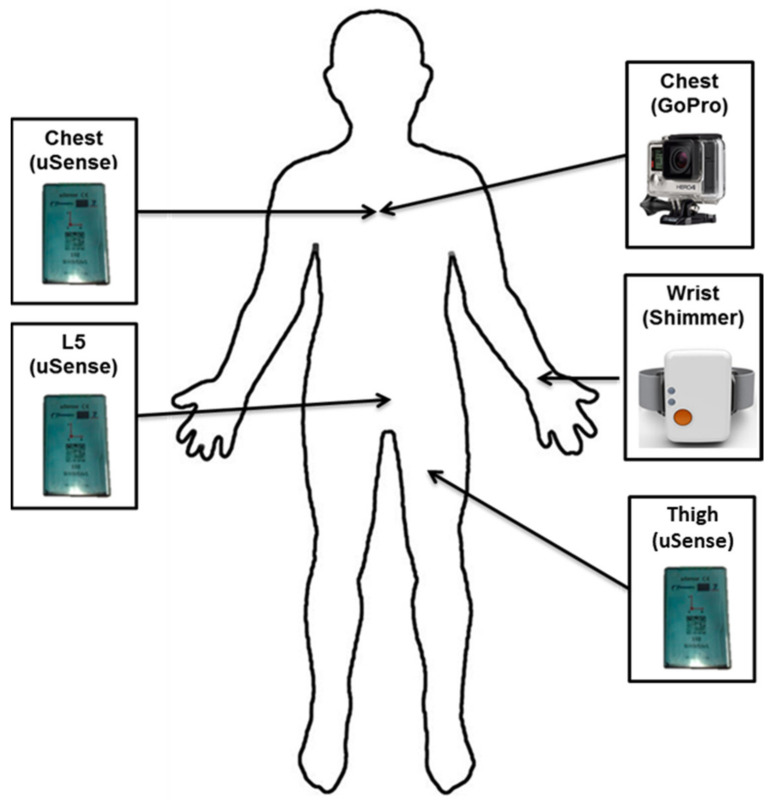
Set of IMU sensors analysed from ADAPT dataset.

The dataset used in this study is a subset of a larger dataset collected by the Department of Neuromedicine and Movement Science, Faculty of Medicine and Health Sciences at the Norwegian University of Science and Technology (NTNU) under the ADAPT project (A Personalized Fall Risk Assessment System for promoting independent living) [37]. The ADAPT dataset was collected in free-living conditions, where the subjects were free to perform ADLs in an unsupervised way. The way of performing activities was natural and unstructured. A total of 20 older adults (76.4 ± 5.6 years) participated in the protocol, performing various ADLs. The subjects were instrumented in the lab (i.e., they wore sensors to record movements and a chest-mounted camera to obtain labels of activities), after which they went home to perform the ADLs in free-living conditions. Subjects were instructed to naturally perform their usual ADLs, but to include a set of defined activities as a part of the free-living protocol, without any instruction or supervision on how to perform them. The activities classified in this work were: sitting, standing, walking, lying. A subset of four of the sensors used in the (out-of-the-lab) free-living protocol from the ADAPT dataset was analysed in this study. The choice of this subset was motivated by the highest performance (F-score) achieved in our earlier work [34]. The subset of sensors is presented in Figure 1, and the sampling frequency of sensors was 100 Hz. The chest-mounted camera shown in Figure 1 served as ground truth [37] to validate the performance (F-score) of sensor-based PAC systems.

Five raters performed the video labelling of the subjects’ movements using the video recordings obtained through the chest-mounted camera, achieving a very high inter-rater reliability of above 90% in labelling the free-living ADLs.

### 2.2. Splitting Training and Testing Data

Each IMU sensor contains six signals (3 for linear acceleration, 3 for angular velocity), resulting in 24 signals. Windows of 5 sec were used, resulting in windows of 500 samples (W). The window length of 5 sec was chosen to maintain consistency with our earlier work [34] and provide comparable results. The N windows were divided into training and testing before developing the ML models and analysing their performance. The data samples of 16 participants out of 20 were used in this study. The data of the remaining 4 participants were not used due to technical issues with the wrist sensor. The dataset of the 16 participants contained a total of 36,139 windows. A data split was performed following the 70%(train)/30%(test)% method (which is one of the common methods to cross-validate the performance of machine learning models). Data from 11 participants were used to train the ML model (N = 26,115 windows), and the remaining data from 5 participants’ (N = 10,024) were used to test the performance of the trained model, as presented in Table 1. The F-score was used as a performance measure for the comparative analysis of PAC systems and will be used interchangeably with performance throughout this study.

### 2.3. Splitting Training and Testing Data

**Figure 2:**
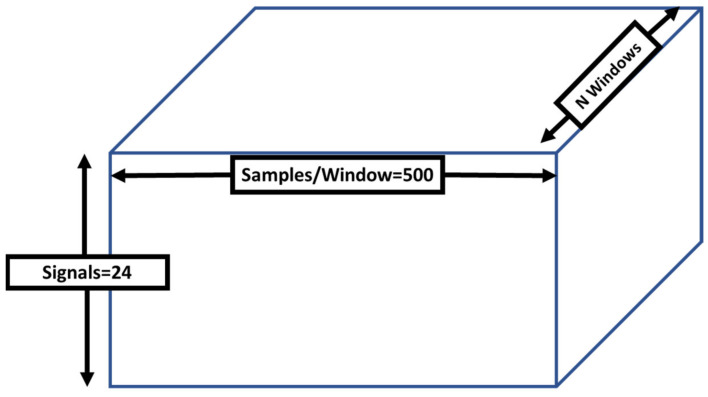
The long short-term memory (LSTM) network’s input data structure.

The LSTM network (a variant of RNN) was used as the deep learning algorithm to develop the PAC system. The LSTM networks were shown to perform better [38] over simple RNNs, due to their ability to remember long-term dependencies of time series data. The LSTM network remembers data dependencies through the explicit memory cells allocated within its architecture and stores information regarding when to keep or forget information from long data sequences. The training data of the four wearable IMU sensors (Figure 1) was fed into the LSTM network. The input data structure is presented in Figure 2. The N windows show the total number of data instances across all participants in the training and testing scenarios (Table 1). The specifications of the proposed LSTM model for the PAC system developed are listed in Table 2.

### 2.4. Classical Machine Learning Algorithm for PAC

The methodology used in this study is the same as the one proposed previously [34]. However, instead of using leave-one-subject-out cross-validation, this study used the training and testing data split presented in Table 1. The performance analysis of classical machine learning-based PAC used the same set of sensors highlighted in Figure 1.

The set of features extracted from the wearable sensors are represented in Table A1 in Appendix A. Three feature selection approaches were used, combined with a weighted SVM classifier to compute the overall performance and performance by class. The feature selection approaches are: correlation-based feature selection (CFS) [42], fast correlation-based filter (FCBF) [43] and ReliefF [44]. The performance of all features, without using any feature selection approach (Table A1, PAC-All-Feat) was also computed.

The F-score was computed as a performance measure to compare the classical machine learning with the deep learning PAC system using the expression below:

$$F_{c}-score=\frac{2*TP_{c}}{2*TP_{c}+FP_{c}+FN_{c}}\times 100$$

where *TP* = True Positive, *TN* = True Negative, *FN* = False Negative, and *FP* = False Positive. The subscript “c” is used to denote class metrics. The overall F-score was calculated by averaging the F-score of all classes.

## 3. Results and Discussion

### 3.1. Performance Analysis of LSTM based PAC System

The LSTM-based PAC system performed well in classifying the ADLs of older people, achieving an overall F-score of 97.23%. The performances by class using the test set for walking, sitting, standing, and lying, as well as overall performances, are presented in Table 3, in which the results of the classical machine learning and deep learning approaches are compared. The respective confusion matrix for the LSTM-based PAC system is shown in Table 4.

It is evident from the findings that the LSTM-based PAC system can classify each ADL with a very high F-score of above 94%, which confirms the strength of deep learning methods. The sitting and lying classes achieved the highest F-score, at around 99%, while the walking and standing classes demonstrated lower scores (94.48% and 96.09%, respectively).

The detailed performance analysis of LSTM-based PAC system using a different sensor combination is presented in Appendix B (see Table A2). It is quite evident from the findings that the LSTM-based PAC system developed using combinations of sensors (two or more) outperformed the single-sensor-based system. A plateau in performance is achieved when three sensors are used, beyond which adding more sensors does not improve performance.

### 3.2. Performance Analysis of Classical Machine Learning Based PAC System

The classification performances obtained through the four scenarios obtained from machine learning-based PAC systems are presented in Table 3, and the corresponding confusion matrices are shown in Table 5. These performances were obtained using the same dataset and train/test data split used for the LSTM-based PAC system reported in Table 1. All the classical machine learning-based PAC systems were able to perform well with an acceptable performance level (F-score > 90%, Table 1). The best performance (F-score) was obtained using the ReliefF-based PAC system; this produced an F-score of 96.83%, which is quite promising and shows the capabilities of the proposed PAC system in classifying ADLs. The second-best performance, of 94.33%, was achieved using all the feature sets. The PAC systems developed on correlation-based feature selection methods, i.e., PAC-CFS and PAC-FCBF, achieved slightly lower F-scores of 93.25% and 91.17%, respectively. To illustrate the impact of feature selection on the PAC system’s performance, the number of features used by each classical machine learning-based PAC system is presented in Table 6. Table 6 shows that CFS and FCBF selected the smallest number of features among all the feature sets analysed and still performed well in classifying the four analysed ADLs. The CFS- and FCBF-based PAC systems used 18 and 17 features, respectively, and the ReliefF-based PAC system used 105 features. The total number of features, without any feature selection approach, was 326. This significant reduction in the feature sets of the correlation-based feature selection methods (CFS, FCBF) could be explained by a slight performance degradation compared to the other two approaches (all-feature set, ReliefF). However, the difference in the performance of these systems was less than 3% and, interestingly, the correlation-based feature selection methods reduced the feature set size up to 94%. The reduction in the feature set can significantly reduce the computational complexity, making the system more feasible and applicable in real-life conditions, which is in line with our earlier findings [34]. The high performance of ReliefF is in line with our earlier analysis [34], where it was shown that ReliefF achieves better performance when the PAC system is implemented over multi-sensor feature sets (which is the scenario in the present study).

### 3.3. Classical Machine Learning Versus Deep Learning: Which Is Better?

**Figure 3:**
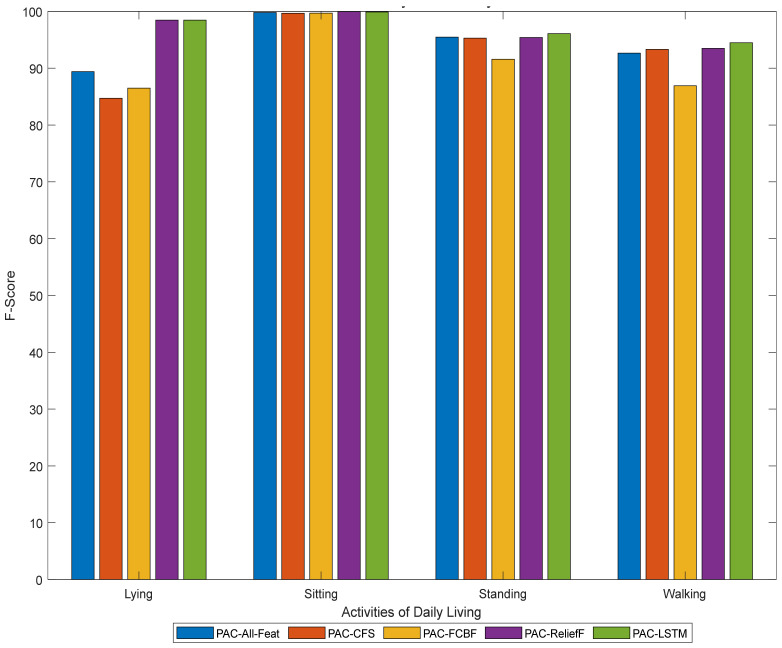
Performance analysis of the classical machine learning and deep learning based PAC systems.

The overall performances obtained through classical machine learning algorithms and LSTM-based deep learning algorithms (see Table 3) suggest that both methodologies can accurately classify the ADLs. The best PAC system obtained in classical machine learning approaches is based on the feature set obtained through ReliefF, and its performance is quite close to the one obtained through deep learning, with a difference of 0.4% (97.23% vs. 96.83%). To get a better insight into class performance, the F-score obtained through all PAC systems is depicted in Figure 3, for both the classical machine learning- and the deep learning-based approaches. All the ADLs, i.e., sitting, standing, walking, and lying, are accurately classified by these PAC systems (PAC-ReliefF, PAC-LSTM) with very high performance by class (above 90%) and the differences in performance among these PAC systems for all classified ADLs are minimal (less than 1%, Table 3—columns 2 and 6).

Moreover, the confusion matrices obtained from the PAC systems (Table 4 and Table 5) suggest that the walking and standing classes are quite often confused with each other in both cases, i.e., in classical machine learning and deep learning, which is the reason for their low F-score. This could be because three out of the four IMU sensors (chest, lower back, and thigh—see Figure 1) have a similar orientation during standing and walking, which could have contributed to this slight degradation in the performance and confusion among the classes. On the contrary, the sitting and lying classes possibly have more distinctive properties, as three out of the four IMU sensors (thigh, chest, lower back) change their orientation from sitting to lying.

Therefore, we can suggest that neither of the approaches, i.e., classical or deep learning, outperformed the other in this work. This result could be related to the fact that a plateau in performance was reached, suggesting that after reaching a certain level of performance, further enhancement might not be possible, regardless of which of the two machine learning approaches is used, as there is a narrow range for improvement and from which to differentiate between the performances of the various PAC systems. Recently, Baldominos et al. [36] performed a similar type of analysis to observe classical machine learning performance versus CNN-based PAC systems (although they analysed the ADLs of younger adults in a constrained environment, rather than in free-living conditions, and they used a CNN instead of an LSTM network). They concluded that the classical machine learning PAC system performed better than the deep learning-based PAC system, which suggests that deep learning methods are not always optimal when referring to wearable sensors based on physical activity classification systems. Their finding is somewhat in line with our present work, as our proposed classical machine learning and deep learning PAC systems performed equally well, with marginal performance difference (<0.4%).

The findings of our study are interesting and show the similar strength of classical and deep learning-based PAC systems in profiling the free-living activities of an elderly population. However, it is essential to mention that the dataset analysed in this study, although quite unique, is not very large, and the nature of the classified activities might not be very challenging in terms of DNNs, as they perform better on larger datasets. PAC systems might behave differently when exploited on datasets from larger cohorts and different populations, with a larger number of activity classes, but this requires further validation in a future study. These observations emphasize that the choice of an appropriate ML algorithm (classical ML or deep learning) depends, to a high degree, on the nature of the problem domain and the quality and the quantity of the labelled dataset. However, it is important to highlight that the dataset used in the study is the first of its kind, in that it included older people in free-living conditions, and underwent an extensive and detailed validation/ground truth annotation process by multiple raters [37]. Moreover, the performance of free-living protocols in the home environment generated more natural patterns and distributions of ADLs than could be obtained in a laboratory-based setup [45]. Future work should focus on exploring other DNNs, such as CNNs or hybrid CNN–LSTMs, or using a temporal CNN as a feature extractor and then feeding the results to a classical ML classifier, such as an SVM.

## 4. Conclusions

This study investigated the performance of classical machine learning-based PAC systems and a deep learning-based PAC system. The dataset used in this study was based on the activities of daily living performed by older people in free-living conditions. There were no constraints on how and when to perform a specific activity, and the participants performed the study protocol in their residential settings. A subset of four wearable inertial sensors from the ADAPT study was analysed in order to classify the daily living activities. The classical machine learning-based PAC system was developed by applying weighted SVM and feature selection. The deep learning-based PAC system was developed using the LSTM approach, by directly feeding in the raw data from the inertial sensors. This study demonstrated that both approaches (classical machine learning and deep learning) can accurately classify the daily living activities of the elderly population with very high performance (F-scores of around 97%). Neither approach was found to be clearly superior to the other, suggesting that both the machine learning and deep learning approaches can classify the activities equally well, in terms of the dataset used in this work.

## Figures and Tables

**Table 1:** Training and testing data split, giving the number of sample windows per participant and classified activity.

Subjects	Walk	Sit	Stand	Lie	Split
1	237	1001	449	54	Testing
2	187	454	119	13	
3	559	1661	801	0	
4	306	2493	406	0	
5	297	593	362	32	
6	493	2078	441	234	Training
7	644	1803	729	19	
8	323	568	495	23	
9	349	1053	554	37	
10	347	1762	654	35	
11	576	617	1503	2	
12	664	836	1293	0	
13	405	1027	589	13	
14	442	1871	774	0	
15	289	711	285	24	
16	222	969	335	27	
Total windows	6340	19,497	9789	513	

Each integer value shows the total number of windows. A single window is equal to 5 sec or 500 data samples, e.g., the Lying class contains 513 windows.

**Table 2:** Specifications of the proposed long short-term memory (LSTM) model for the physical activity classification (PAC).

Parameter	Value
Window Size	N (500 samples)
Sampling frequency	100 Hz
Number of features/signals	24 (F)
Training data feature space	(26,115, 500, 24)
Training data label space	(26,115, 1)
Testing data feature space	(10,024, 500, 24)
Training data label space	(10,024, 1)
Cost Function	Softmax Cross Entropy [39]
Optimizer	Adam Optimizer [40]
LSTM Layers	2
**No of Hidden Units**	32
**Activation function**	ReLU [41]
**Regularization**	L2 regularization
**Learning rate**	0.0025
**Batch size**	1500
**Loss function**	Softmax cross entropy with logits
**Software used**	Tensflow with GPU
**System used**	Lenovo Legion 5 Ryzen 7 16GB 512GB SSD RTX 2060 15.6” Win10 Home Gaming Laptop

LSTM = long short-term memory; ReLU = Rectified Linear Unit.

**Table 3:** Performance analysis of different PAC systems using the test set.

ADLs Classified	PAC-LSTM (%)	PAC-All-Feat (%)	PAC-CFS (%)	PAC-FCBF (%)	PAC-ReliefF (%)
Walking	94.48	92.65	93.32	86.91	93.48
Sitting	99.90	99.81	99.68	99.69	99.95
Standing	96.09	95.48	95.29	91.58	95.41
Lying	98.46	89.39	84.72	86.49	98.46
Overall F-score	97.23	94.33	93.25	91.17	96.83

ADL = activities of daily living; PAC = physical activity classification; LSTM = long short-term memory; CFS = correlation-based feature selection; FCBF = fast correlation-based filter.

**Table 4:** Confusion matrix obtained using the proposed LSTM-based PAC system using the test set.

	Predicted Class				
**Actual Class**		**Walking**	**Sitting**	**Standing**	**Lying**
	**Walking**	1464	3	118	0
	**Sitting**	2	6197	3	0
	**Standing**	48	1	2088	0
	**Lying**	0	3	0	96

**Table 5:** Confusion matrix of the PAC system using (a) all features (b) CFS (c) FCBF (d) ReliefF.

	(a)→ All features					(b)→CFS				
	Predicted class					Predicted class				
**Actual class**		**Walking**	**Sitting**	**Standing**	**Lying**		**Walking**	**Sitting**	**Standing**	**Lying**
	**Walking**	1449	1	136	0	**Walking**	1424	0	162	0
	**Sitting**	18	6181	3	0	**Sitting**	1	6164	4	33
	**Standing**	58	0	2079	0	**Standing**	41	0	2096	0
	**Lying**	17	2	0	80	**Lying**	0	2	0	97
	**(c)**→ **FCBF**					**(d)**→ **ReliefF**				
	**Predicted class**					**Predicted class**				
**Actual class**		**Walking**	**Sitting**	**Standing**	**Lying**		**Walking**	**Sitting**	**Standing**	**Lying**
	**Walking**	1268	0	318	0	**Walking**	1442	1	143	0
	**Sitting**	4	6167	4	27	**Sitting**	1	6200	1	0
	**Standing**	60	0	2077	0	**Standing**	56	0	2081	0
	**Lying**	0	3	0	96	**Lying**	0	3	0	96

**Table 6:** Number of features used in the classical machine learning based PAC system.

Feature Selection Technique	Number of Features
All features (no feature selection)	326
CFS	18
FCBF	17
ReliefF	105

## Data Availability

The script from the ADAPT validation data set used in this study is available on request to Jorunn L. Helbostad (jorunn.helbostad@ntnu.no).

## Appendix A

Features Used in Classical Machine Learning based PAC System

**Table A1:** Features Computed from Each Signal.

Feature #	Feature Description	Feature #	Feature Description
1–3	Mean of acceleration (x, y, z) ^a^	40–42	Variance of angular velocity (x, y, z)
4–6	Variance of acceleration (x, y, z)	43–45	Correlation between axes of angular velocity (x, y, z)
7–9	Correlation between axes of acceleration (x, y, z)	46–48	Energy of angular velocity (x, y, z)
10–12	Energy of body acceleration (BA) component (x, y, z)	49	SMA of the angular velocity
13	Signal magnitude area (SMA) of BA component	50	Mean of MV of angular velocity
14	Tilt angle obtained from gravitational acceleration (GA) component in vertical direction	51	Variance of MV of angular velocity
15–17	Mean of GA components (x, y, z)	52	Energy of MV of angular velocity
18	Mean of magnitude vector (MV) of BA component	53–55	Mean of jerk signal from angular velocity (x, y, z)
19	Variance of MV of BA component	56–58	Variance of jerk signal from angular velocity (x, y, z)
20	Energy of MV of BA component	59–61	Correlation between the axes of the jerk signal from angular velocity (x, y, z)
21–23	Mean of jerk signal from acceleration (x, y, z)	62–64	Energy of jerk signal from angular velocity (x, y, z)
24–26	Variance of jerk signal from acceleration (x, y, z)	65	SMA of the jerk signal from angular velocity
27–29	Correlation between the axes of jerk signal from acceleration (x, y, z)	66	Mean of MV of jerk signal from angular velocity
30–32	Energy of the jerk signal from acceleration (x, y, z)	67	Variance of MV of jerk signal from angular velocity
33	SMA of the jerk signal from acceleration	68	Energy of MV of jerk signal from angular velocity
34	Mean of MV of jerk signal from acceleration	69–71 ^b^	Attenuation constant between sensor combinations of acceleration (x, y, z)
35	Variance of MV of jerk signal from acceleration	72–74 ^b^	Correlation between sensor combinations of acceleration (x, y, z)
36	Energy of MV of jerk signal from acceleration	75–77 ^b^	Correlation between sensor combinations of angular velocity signal (x, y, z)
37–39	Mean of angular velocity (x, y, z)		

^a^ x, y, z show that all three axes of the signal (can be raw acceleration, BA component, angular velocity, jerk etc.) are used to compute the respective features. ^b^ Features from 69–74 were considered only if a sensor combination was analyzed.

## Appendix B

Performance Analysis of LSTM based PAC System on Test Set

**Table A2:** Performance analysis (F-score) of LSTM-based PAC system using different sensor locations.

Sensor Combination	Walking	Sitting	Standing	Lying	Overall F-Score
L5	85.2 %	95.0 %	80.7 %	76.9 %	84.4 %
Wrist (W)	71.5 %	89.7 %	64.2 %	0.0 %	56.3 %
Thigh (T)	95.0 %	99.1 %	96.4 %	0.0 %	72.6 %
Chest (C)	71.4 %	86.1 %	61.7 %	98.5 %	79.4 %
T + L5	94.2 %	99.6 %	95.9 %	81.4 %	92.8 %
T + C + L5	94.2 %	99.9 %	95.9 %	99.5 %	97.3 %
T + C + L5 + W	94.5	99.9	96.1	98.5	97.2

## References

[1] World Health Organization (2015). Global Recommendations on Physical Activity for Health—2010.

[2] McPhee J.S., French D.P., Jackson D., Nazroo J., Pendleton N., Degens H. (2016). Physical activity in older age: Perspectives for healthy ageing and frailty. Biogerontology.

[3] Bangsbo J., Blackwell J., Boraxbekk C.-J., Caserotti P., Dela F., Evans A.B., Jespersen A.P., Gliemann L., Kramer A., Lundbye-Jensen J. (2019). Copenhagen Consensus statement 2019: Physical activity and ageing. Br. J. Sports Med..

[4] European Commission (2012). The 2012 Ageing Report: Economic and budgetary projections for the EU-27 Member States (2010–2060). https://ec.europa.eu/economy_finance/publications/european_economy/2012/pdf/ee-2012-2_en.pdf.

[5] Preece S.J., Goulermas J.Y., Kenney L.P., Howard D. (2008). A comparison of feature extraction methods for the classi-fication of dynamic activities from accelerometer data. IEEE Trans. Biomed. Eng..

[6] Figo D., Diniz P.C., Ferreira D.R., Cardoso J.M. (2010). Preprocessing techniques for context recognition from accel-erometer data. Pers. Ubiquitous Comput..

[7] Anguita D., Ghio A., Oneto L., Parra F.X.L., Ortiz J.L.R. (2012). Human Activity Recognition on Smartphones Using a Multiclass Hardware-Friendly Support Vector Machine. International Workshop on Ambient Assisted Living.

[8] Staudenmayer J., He S., Hickey A., Sasaki J., Freedson P.S. (2015). Methods to estimate aspects of physical activity and sedentary behavior from high-frequency wrist accelerometer measurements. J. Appl. Physiol..

[9] Awais M., Palmerini L., Chiari L. Physical activity classification using body-worn inertial sensors in a multi-sensor setup. Proceedings of the 2016 IEEE 2nd International Forum on Research and Technologies for Society and Industry Leveraging a better tomorrow (RTSI).

[10] Kwon M.-C., Choi S. (2018). Recognition of Daily Human Activity Using an Artificial Neural Network and Smartwatch. Wirel. Commun. Mob. Comput..

[11] O’Mahony N., Campbell S., Carvalho A., Harapanahalli S., Velasco-Hernández G., Krpalkova L., Riordan D., Walsh J. (2019). Deep Learning vs. Traditional Computer Vision.

[12] LeCun Y., Bengio Y., Hinton G. (2015). Deep learning. Nature.

[13] Hochreiter S., Schmidhuber J. (1997). Long short-term memory. Neural Comput..

[14] LeCun Y., Boser B., Denker J., Henderson D., Howard R., Hubbard W., Jackel L. (1990). Handwritten digit recognition with a back-propagation network. Advances in Neural Information Processing Systems.

[15] El Hihi S., Bengio Y. Hierarchical recurrent neural networks for long-term dependencies. Proceedings of the Advances in Neural Information Processing Systems.

[16] Rajkomar A., Oren E., Chen K., Dai A.M., Hajaj N., Hardt M., Liu P.J., Liu X., Marcus J., Sun M. (2018). Scalable and accurate deep learning with electronic health records. npj Digit. Med..

[17] Ahmad T., Chen H. (2019). Deep learning for multi-scale smart energy forecasting. Energy.

[18] Tian Y., Pei K., Jana S., Ray B. Deeptest: Automated testing of deep-neural-network-driven autonomous cars. Proceedings of the 40th International Conference on Software Engineering.

[19] Amodei D., Ananthanarayanan S., Anubhai R., Bai J., Battenberg E., Case C., Caspe J., Catanzaro B., Cheng Q., Chen G. Deep speech 2: End-to-end speech recognition in english and mandarin. Proceedings of the International Conference on Machine Learning.

[20] Heaton J., Polson N., Witte J.H. (2017). Deep learning for finance: Deep portfolios. Appl. Stoch. Models Bus. Ind..

[21] Bhattacharya S., Lane N.D. From smart to deep: Robust activity recognition on smartwatches using deep learning. Proceedings of the 2016 IEEE International Conference on Pervasive Computing and Communication Workshops (PerCom Workshops).

[22] Ordóñez F.J., Roggen D. (2016). Deep Convolutional and LSTM Recurrent Neural Networks for Multimodal Wearable Activity Recognition. Sensors.

[23] Ronao C.A., Cho S.-B. (2016). Human activity recognition with smartphone sensors using deep learning neural networks. Expert Syst. Appl..

[24] Morales J., Akopian D. (2017). Physical activity recognition by smartphones, a survey. Biocybern. Biomed. Eng..

[25] Gochoo M., Tan T.-H., Liu S.-H., Jean F.-R., Alnajjar F., Huang S.-C. (2018). Unobtrusive Activity Recognition of Elderly People Living Alone Using Anonymous Binary Sensors and DCNN. IEEE J. Biomed. Heal. Informatics.

[26] Hassan M.M., Huda S., Uddin Z., Almogren A., Alrubaian M.A. (2018). Human Activity Recognition from Body Sensor Data using Deep Learning. J. Med Syst..

[27] Hassan M.M., Uddin Z., Mohamed A., Almogren A. (2018). A robust human activity recognition system using smartphone sensors and deep learning. Futur. Gener. Comput. Syst..

[28] Pienaar S.W., Malekian R. Human Activity Recognition using LSTM-RNN Deep Neural Network Architecture. Proceedings of the 2019 IEEE 2nd Wireless Africa Conference (WAC).

[29] Lawal I.A., Bano S. Deep human activity recognition using wearable sensors. Proceedings of the Proceedings of the 12th ACM International Conference on PErvasive Technologies Related to Assistive Environments.

[30] Wang H., Zhao J., Li J., Tian L., Tu P., Cao T., An Y., Wang K., Li S. (2020). Wearable Sensor-Based Human Activity Recognition Using Hybrid Deep Learning Techniques. Secur. Commun. Networks.

[31] Papagiannaki A., Zacharaki E.I., Kalouris G., Kalogiannis S., Deltouzos K., Ellul J., Megalooikonomou V. (2019). Rec-ognizing physical activity of older people from wearable sensors and inconsistent data. Sensors.

[32] Aicha A.N., Englebienne G., Van Schooten K.S., Pijnappels M., Kröse B. (2018). Deep Learning to Predict Falls in Older Adults Based on Daily-Life Trunk Accelerometry. Sensors.

[33] Awais M., Palmerini L., Bourke A.K., Ihlen E.A.F., Helbostad J.L., Chiari L. (2016). Performance Evaluation of State of the Art Systems for Physical Activity Classification of Older Subjects Using Inertial Sensors in a Real Life Scenario: A Benchmark Study. Sensors.

[34] Awais M., Chiari L., Ihlen E.A.F., Helbostad J.L., Palmerini L. (2018). Physical Activity Classification for Elderly People in Free-Living Conditions. IEEE J. Biomed. Heal. Informatics.

[35] Shakya S.R., Zhang C., Zhou Z. (2018). Comparative Study of Machine Learning and Deep Learning Architecture for Human Activity Recognition Using Accelerometer Data. Int. J. Mach. Learn. Comput..

[36] Baldominos Gómez A., Cervantes A., Sáez Achaerandio Y., Isasi P. (2019). A Comparison of Machine Learning and Deep Learning Techniques for Activity Recognition using Mobile Devices. Sensors.

[37] Bourke A., Ihlen E.A.F., Bergquist R., Wik P.B., Vereijken B., Helbostad J.L. (2017). A Physical Activity Reference Data-Set Recorded from Older Adults Using Body-Worn Inertial Sensors and Video Technology—The ADAPT Study Data-Set. Sensors.

[38] Graves A., Mohamed A.-R., Hinton G. Speech recognition with deep recurrent neural networks. Proceedings of the 2013 IEEE International Conference on Acoustics, Speech and Signal Processing.

[39] Softmax Cross Entropy. https://www.tensorflow.org/api_docs/python/tf/nn/softmax_cross_entropy_with_logits.

[40] Adam Optimizer. https://www.tensorflow.org/versions/r1.15/api_docs/python/tf/keras/optimizers/Adam.

[41] Rectified Linear Unit. https://www.tensorflow.org/api_docs/python/tf/nn/relu.

[42] Hall M.A., Smith L.A. Feature selection for machine learning: Comparing a correlation-based filter approach to the wrapper. Proceedings of the 12th International FLAIRS Conference.

[43] Yu L., Liu H. Feature selection for high-dimensional data: A fast correlation-based filter solution. Proceedings of the 20th International Conference on Machine Learning (ICML-03).

[44] Moncada-Torres A., Leuenberger K., Gonzenbach R., Luft A., Gassert R. (2014). Activity classification based on inertial and barometric pressure sensors at different anatomical locations. Physiol. Meas..

[45] Awais M., Mellone S., Chiari L. Physical Activity Classification Meets Daily Life: Review on Existing Methodologies and Open Challenges. Proceedings of the 37th Annual International Conference of the IEEE Engineering in Medicine and Biology Society (EMBC).

